# First Description of Atypical *Aspergillus floccosus* as Cause of Canine Systemic Aspergillosis With Discospondylitis

**DOI:** 10.1002/vms3.70466

**Published:** 2025-06-14

**Authors:** Talita Bordoni, Samuel Okonji, Filippo Maria Dini, Monica Cricca, Gualtiero Gandini, Monica Caffara, Roberta Galuppi

**Affiliations:** ^1^ Department of Veterinary Medical Sciences Alma Mater Studiorum – University of Bologna Bologna Italy; ^2^ Department of Medical and Surgical Sciences Alma Mater Studiorum – University of Bologna Bologna Italy

**Keywords:** *Aspergillus floccosus*, canine aspergillosis, cryptic species, dog, disseminated mould infection

## Abstract

Disseminated aspergillosis is uncommon in dogs and typically caused by *Aspergillus terreus*. This report describes a systemic aspergillosis linked to discospondylitis in an 8‐year‐old spayed female German Shepherd presented with a 2‐week history of neck pain, progressive proprioceptive ataxia and ambulatory paraparesis and lameness of the left front limb. For the first time, an atypical strain of *Aspergillus floccosus*, section *Terrei*, was identified by culture and molecular methods. This finding emphasises the importance of molecular biology in fungal species identification, which is essential for accurate diagnosis and appropriate treatment. Our report further highlights that fungal infections should always be considered in German Shepherds with neurological signs, and the importance of maintaining antifungal therapy even in patients with few or no clinical or laboratory signs of active infection to prevent deterioration and death.

## Graphical abstract

A case of disseminated aspergillosis in dog was described due to an atypical strain of *Aspergillus floccosus*. Aspergillosis should be considered in the differential diagnosis of dogs with neurological signs and nonspecific changes in CBC or CMP, particularly in German Shepherd dogs. Molecular identification is crucial for an appropriate diagnosis of cryptic *Aspergillus* species. Finally, the importance of maintaining antifungal therapy was underlined.

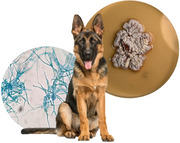

## 1 Introduction

Disseminated mould infections in dogs can be caused by several etiological agents including hyaline and dematiaceous hyphomycetes; amongst them, the most frequently involved belong to the genus *Aspergillus* (Elad et al. 2019). Systemic canine aspergillosis is a severe infectious disease caused by fungi of the genus *Aspergillus*, often leading to fatal outcomes (Corrigan et al. 2016). German Shepherds are the most affected breed (Schultz et al. 2008) due to an inherited mucosal immune deficiency (Bennett et al. 2018; Zhang et al. 2012), although other breeds may be affected, particularly if immunocompromised or with comorbidities (Burrough et al. 2012). The primary route of entry for *Aspergillus* spp. is typically the respiratory tract, but wounds and exposed bone trauma are also potential risk factors (Del Magno et al. 2022). Haematogenous spread can lead to multiple organ involvement, particularly the respiratory, gastrointestinal, nervous, musculoskeletal, urinary, lymphatic systems and eyes (Garcia et al. 2012; Schultz et al. 2008). The clinical presentation in canine species is highly variable, both in terms of severity and anatomical distribution, with symptoms that may emerge acutely or follow a chronic course, as reported in several studies (Zhang et al. 2012; Bennett et al. 2018). Early stages are typically characterised by nonspecific signs such as fever, fatigue, weight loss and lymph node enlargement, which tend to worsen as the disease advances. *Aspergillus terreus* is the most frequently reported species involved in canine systemic mycosis (Kabay et al. 1985, Lim et al. 2022) although occasionally *Aspergillus versicolor* (Zhang et al. 2012), *Aspergillus alabamensis* (Burrough et al. 2012) and *Aspergillus deflectus* (Bennett et al. 2018; Robinson et al. 2000) are also reported. The present study describes the first case of canine aspergillosis caused by *Aspergillus floccosus*, with the isolation of an atypical phenotype.

## 2 Case Report

An 8‐year‐old spayed female German Shepherd was presented with a 2‐week history of progressive pelvic limbs gait abnormalities associated with left forelimb lameness and severe neck pain.

General physical examination was unremarkable, while neurological examination showed low head carriage and proprioceptive ataxia of both pelvic limbs, and mild ambulatory paraparesis and Grade II lameness of the left forelimb. Proprioceptive positioning was decreased on both hindlimbs and on the left front limb. Severe reduction of the withdrawal reflex was also present on the left forelimb. Neck manipulation was accompanied by severe pain.

Based on the neurological examination, a left lateralised C6‐T2 myelopathy with a presumed root sign on the left forelimb was suspected. The main differential diagnoses included an inflammatory/infectious, degenerative (such as an intervertebral disc herniation) or neoplastic disease.

Complete blood count (CBC) and comprehensive metabolic panel (CMP) revealed only generic signs of systemic inflammation (C‐reactive protein [CRP] 4.44, range, 0–0.8 mg/dL). Chest x‐rays were within normal limits, whereas cervicothoracic and thoracic spinal radiographs showed multiple narrowing of the intervertebral discs at the level of C6–C7, C7–T1, T5–T9, T10–T11 and T12–T13, associated with irregular endplate profile and sclerosis of the subchondral bone, consistent with multifocal inflammatory/infectious discospondylopathy.

**FIGURE 1:**
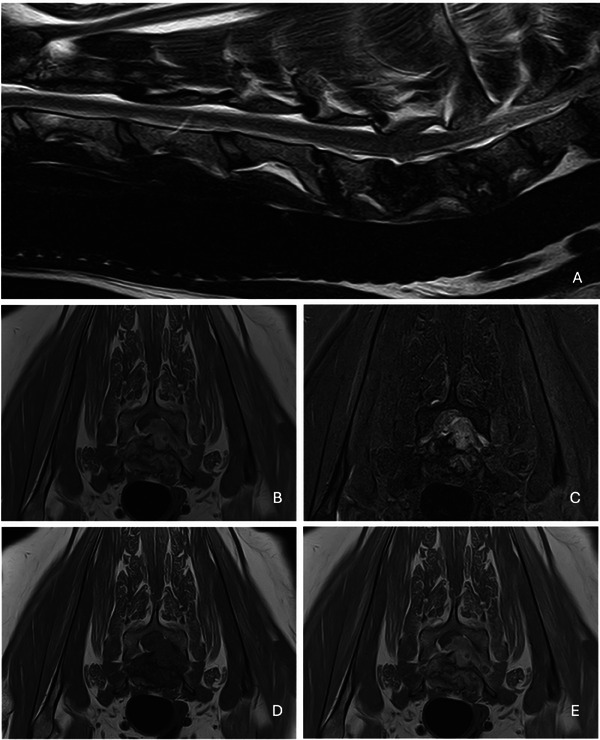
MRI of the cervical and cervico‐thoracic spinal cord showing multiple discospondylitis associated with spinal epidural empyema: (A) sagittal t2‐w sequence; (B) transverse t2‐w sequence; (C) transverse stir; (D) transverse t1‐w sequence; (E) transverse t1‐w post‐contrast sequence. Severe and diffuse changes in signal intensity of the intervertebral discs and adjacent end‐plate at the level of C5–T1 with C6–C7 T2 hyperintense compressive extradural material consistent with spinal epidural empyema.

Magnetic resonance imaging (MRI) of the cervical and cervicothoracic spinal cord was performed to better define the lesions seen on radiographs. MRI confirmed the suspicion of alteration of multiple intervertebral discs showing severe and multiple signal intensity abnormalities with end‐plates erosion, multiple areas of hyperintensity signal on T2W of the intervertebral discs (from C5 to T1) and a left ventrolateral C6–C7 compressive myelopathy due to T2W hyperintense, T1W isointense and contrast‐enhancing space occupying lesion associated with a compression of the left C7 nerve root suggestive of spinal epidural empyema (Figure 1).

Biopsy of the infected disc material was refused by the owner and, since fungal discospondylitis was included in the list of the differential diagnoses, urine was collected to detect fungal hyphae, as the examination of the urinary sediment has proven to be highly indicative and minimally invasive in cases of systemic fungal diseases (Del Magno et al. 2022; Lim et al. 2022).

**FIGURE 2:**
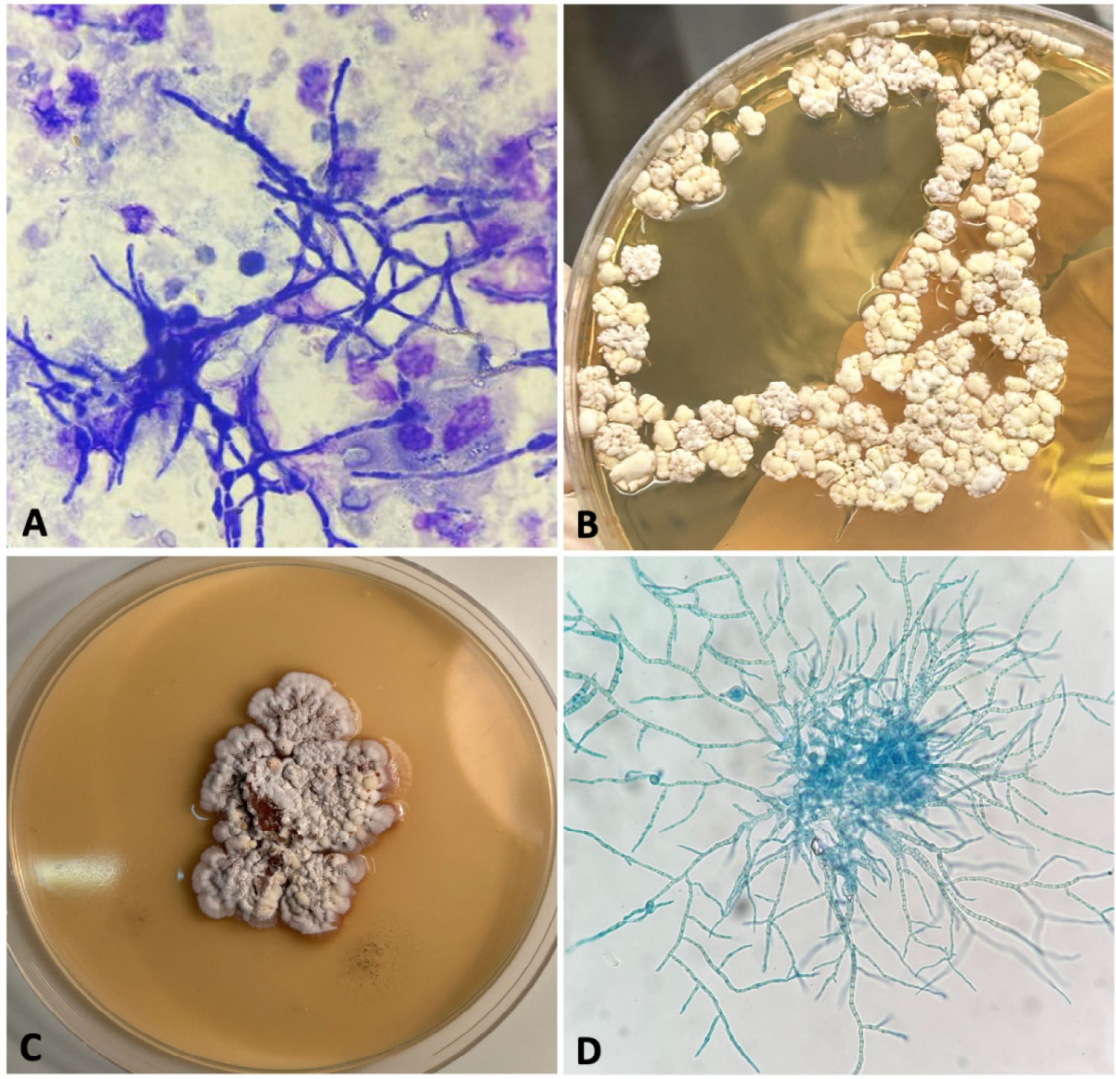
(A) MGG staining of urinary sediment revealing numerous hyphae (magnification 400×); (B) colonies isolated from urinary sediment on SAB‐CAF after 15 days of incubation at 26°C; (C) Macroscopic appearance of the colonies subcultured on CZAPEK agar after 20 day of incubation at 26°C; (D) Lactophenol blue staining revealing only hyphae and chlamydospores, without typical conidial heads (magnification 400×).

The fast microscopic examination of the May‐Grünwald Giemsa (MGG)‐stained urinary sediment revealed the presence of short hyphae (Figure 2A), demonstrating the subclinical involvement of the urinary tract. Culture was performed on Difco Sabouraud Dextrose Agar medium (BD) with 0.05 mg/L chloramphenicol (Sigma) (SAB‐CAF) at 26°C and 37°C for 10 days.

Due to the presence of fungal elements in the urine sediment, fluconazole (Fluconazole; EG S.p.a.) 5 mg/kg twice daily was chosen as antifungal therapy according to the suggestion of the internal medicine colleagues. In addition, analgesic therapy with gabapentin (Gabapentin, Teva Italia S.r.l.) 10 mg/kg three times a day and tramadol (Altadol, Formevet S.r.l.) 3 mg/kg also three times daily was administered. At the 2‐week follow‐up, neurological signs were significantly improved with resolution of pain and improvement of hindlimb proprioceptive ataxia and left forelimb lameness. CRP value was within normal limits, and urinalysis showed no fungal hyphae.

The first culture, periodically examined, macroscopically exhibited the presence of slow growing colonies with rugose appearance (Figure 2B) also when subcultured on Difco Czapeck Solution Agar (BD) medium (Figure 2C). Microscopic observation revealed the presence of hyphae containing numerous terminals and intercalary chlamydospores, but no conidial structures were observed (Figure 2D). The grown colonies were processed for DNA extraction, using Pure Link Genomic DNA Mini kit (Invitrogen by Thermo Fisher), according to the manufacturer's instructions. PCR for ITS region was performed (White et al. 1990), following to the protocol of Schoch et al. (2012). Sequence of the amplicon, compared with published data by BLAST tools, allowed to identify the isolate as belonging to the genus *Aspergillus*, with 100% identity with *A. terreus* (MT529973) and 99.84 % with *A. floccosus* (OL711796).

Three months later, while still on fluconazole treatment, haematuria was detected. Urinalysis showed the presence of fungal hyphae and the same fungus was isolated from the cultures. Antifungal susceptibility testing for fluconazole and itraconazole were performed according to EUCAST method, despite the lack of conidia. Results revealed MIC values higher than 256 mg/L and equal to 0.25 mg/L for fluconazole and itraconazole, respectively. Treatment with itraconazole (Itraconazole, EG S.p.a.) at 5 mg/kg twice daily was then started and fluconazole suspended. The condition of the dog significantly improved with complete disappearance of proprioceptive ataxia on the hindlimbs and lameness of the left front limb.

**FIGURE 3:**
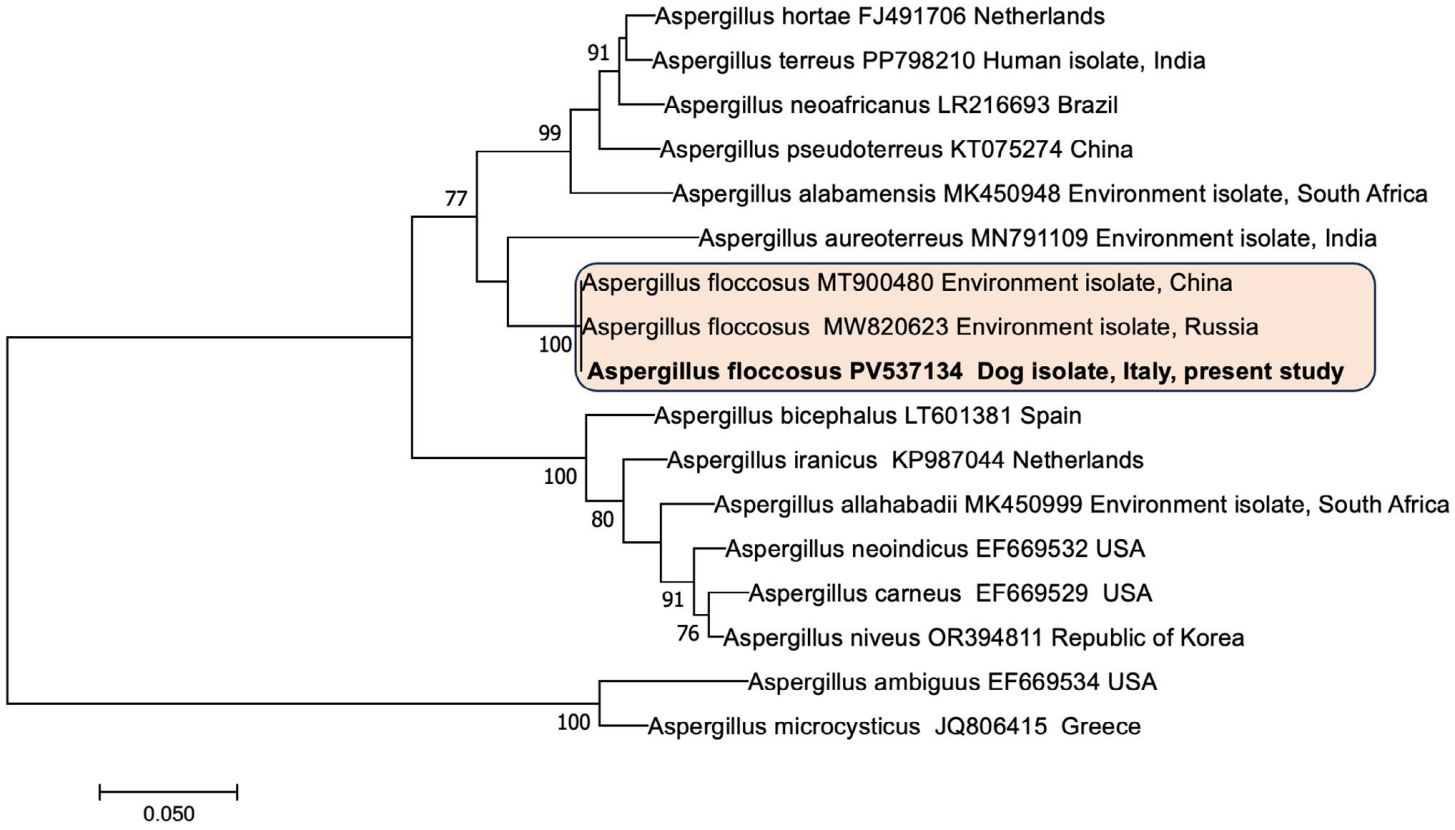
Phylogenetic tree constructed using maximum likelihood method based on the Kimura 2‐parameter model. The tree is drawn to scale, with branch lengths measured in the number of substitutions per site. The analysis involved 17 nucleotide sequences. All positions containing gaps and missing data were eliminated. There were a total of 371 positions in the final dataset. Evolutionary analyses were conducted in MEGA7.

In order to perform accurate species identification, sequencing of beta‐tubulin gene was conducted according to Burrough et al. (2012), using the primers described by Romanelli et al. (2014). The beta‐tubulin sequence obtained compared with published data by BLAST, showed 100% similarity to *A. floccosus* (MH292819) and 99.81% with *A. terreus* (LR693830). Sequence alignments were performed using BioEdit 7.2.5, maximum composite likelihood (MCL) tree (K2P model and bootstrap of 1000 replicates) was obtained using MEGA 7 (Figure 3) (Kimura 1980; Kumar et al. 2016).

The beta‐tubulin gene sequence generated in this study has been deposited in GenBank under accession number PV537134.

After 16 months from presentation, the dog was referred with acute and severe spastic non‐ambulatory paraparesis in association with pain on palpation at the level of the last thoracic vertebrae. A T3‐L3 myelopathy was suspected. The anamnesis revealed that antifungal therapy had been discontinued 2 weeks earlier by the owner without any medical indication.

CBC revealed mild neutrophilic leukocytosis (17,210 leukocytes [range 5000–14,000/mm^3^] and 15,060 neutrophils [range 3000–10,000/mm^3^]), while CMP showed a mild increase in CRP to 1.36 mg/dL [range 0–0.8 mg/dL].

Fungal hyphae were found again in the urine sediment.

**FIGURE 4:**
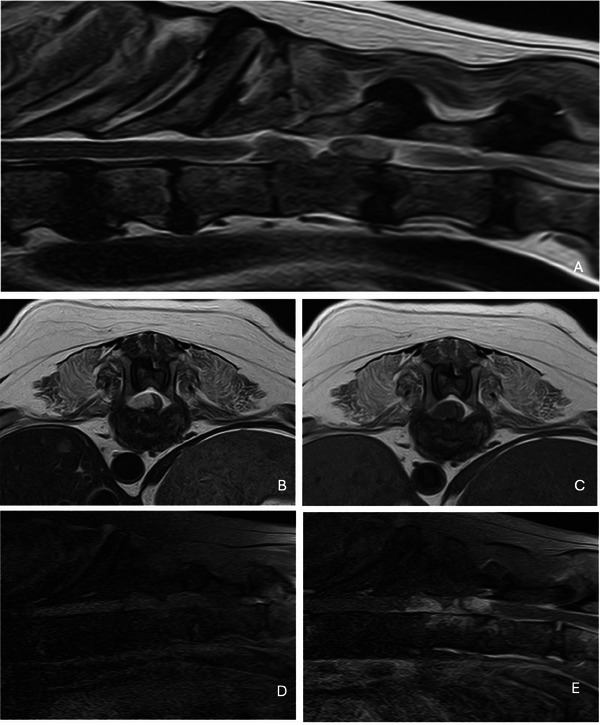
MRI of the thoraco‐lumbar spinal cord performed 16 months from presentation: (A) sagittal t2‐w sequence; (B) transverse t2‐w sequence; (C) transverse t1‐w sequence; (D) transverse t1‐w fat sat sequence; (E) TRANSVERSE T1‐W FAT sat post‐contrast sequence. Multiple irregular and lytic changes of the adjacent end‐plates and loss of the normal intervertebral discs morphology. There is also a severe T11‐T13 extradural compressive myelopathy caused by high‐uptake contrastographic material consistent with spinal epidural empyema.

A second MRI of the thoracolumbar spine revealed severe right‐sided compressive T11‐T13 myelopathy caused by a suspected empyema associated with significant changes in shape and signal intensity of various intervertebral discs and their respective endplates at the thoracolumbar level (Figure 4).

Euthanasia was performed due to the rapid worsening of neurological condition leading to paraplegia in the subsequent 48 h.

## 3 Discussion

Canine disseminated aspergillosis is a rare but severe condition, frequently resulting in fatal outcome (Corrigan et al. 2016; Zhang et al. 2012). In Italy, reported cases are few (Del Magno et al. 2022; Sforna et al. 2011) but the data is most probably underestimated. Due to highly variable clinical signs and limited knowledge of this mycosis, diagnosis may be difficult (Del Magno et al. 2022; Schultz et al. 2008; Zhang et al. 2012) and commonly reached when the clinical condition is already advanced. Clinical signs of discospondylitis are often nonspecific and highly variable including lethargy, anorexia, weight loss, fever and frequent signs of spinal pain (Harris et al. 2013). Affected dogs may be neurologically normal, or they may have ambulatory deficits secondary to spinal cord compression due to empyema, vertebral subluxation or pathological fracture (Feldenzer et al. 1987; Monteiro et al. 2016).

Although blood tests represent an initial screening in patients with suspected discospondylitis and CRP seems to represent useful biomarker in the diagnosis (Nye et al. 2020), diagnostic imaging is essential for identification of the infection of the intervertebral disc. Radiography is a first‐step diagnostic imaging approach, being non‐invasive, inexpensive and readily available in most veterinary practices (Ruoff et al. 2018). However, it may not be able to detect changes in the early stages of the disease. Therefore, advanced diagnostic imaging such as MRI or computed tomography (CT) is required to confirm the diagnosis (Shamir et al. 2001). Specifically, MRI is the gold standard for the diagnosis of discospondylitis (Harris et al. 2013) and offers distinct advantages over the other imaging modalities. Due to its high soft tissue resolution, MRI can better define the inflammation of the intervertebral disc and surrounding tissues and detect the presence of spinal empyema, providing essential information for treatment planning (De Stefani et al. 2008).

Beside the advanced imaging findings, laboratory investigation is crucial to confirm the presence of the fungus. This case demonstrates that urine specimens can be highly informative while being minimally invasive. They provide rapid, preliminary data that can help define the next steps in the diagnostic work‐up. Molecular confirmation of the pathogen is crucial for appropriate treatment, especially in cases where the morphological presentation is atypical (Lackner et al. 2019). In this case, the phenotypic traits in culture were not indicative for *Aspergillus* sp. The pathogen was identified molecularly as *A. floccosus*, a species not previously reported as a causative agent in animals or humans. It was described for the first time by Samson et al. (2011) and has only been isolated from environmental matrices (Pangging et al. 2022; Samson et al. 2011). The belonging of *A. floccosus* at the section *Terrei* (Lackner et al. 2019) confirms that cases of canine systemic aspergillosis are frequently caused by species included in this section (Kabay et al. 1985; Lim et al. 2022; Schultz et al. 2008). The unusual morphology of the fungus made identification challenging. In fact, we observed an atypical presentation on both SAB‐CAF and CZAPEK agar compared to the description of *A. floccosus* by Samson et al. (2011) and Pangging et al. (2022), who reported a floccose texture and the presence of typical, densely columnar conidial heads. In our culture, no characteristic *Aspergillus* heads were observed but only chlamydospores. To our knowledge, the reason for the different morphology is unclear. Overall, the morphological diagnosis of *Aspergillus* species can be quite complex due to factors such as the presence of cryptic species and the lack of characteristic microscopic structures. The phenotypic traits of fungi within the *Aspergillus* genus are often unstable, and clinical isolates frequently present atypically, with minimal or no sporulation (Balajee et al. 2007). For instance, Bennett et al. (2018) described fungal colonies that grew without the typical appearance of *Aspergillus* spp. Similarly, Burrough et al. (2012) reported a case similar to ours, in which *A. alabamensis* isolated from disseminated aspergillosis in a dog showed extremely slow growth and unusual phenotypic features, without conidial formation. For the abovementioned reasons, molecular identification of *Aspergillus* spp. is highly recommended, using the ribosomal internal transcribed spacer region for genus identification and protein‐coding genes, such as beta‐tubulin, for precise species determination (Balajee et al. 2007).

Therapeutic management of this type of invasive infection, which is usually lifelong, is another key issue. A serious problem is the high cost of last generation antimycotic drugs, such as voriconazole and posaconazole, which limits the available treatments for animals (Corrigan et al. 2016; Koehler et al. 2013). In addition, the prognosis for canine aspergillosis is usually poor even when a specific therapy is administered (Morabito et al. 2020; Schultz et al. 2008). Survival of dogs with systemic aspergillosis can vary widely (Schultz et al. 2008), with a median survival time of 226 days for treated dogs (Lim et al. 2022). However, in some cases, survival has exceeded 1000 days, as reported by Kelly et al. (1995) and Bennet et al. (2018), while often a rapid deterioration until death is observed after interruption of treatment (Del Magno et al. 2022; Schultz et al. 2008), as in our case.

## 4 Conclusions

In conclusion, to the best of the authors' knowledge, this is the first description of canine disseminated aspergillosis with discospondylitis caused by *A. floccosus*. This case highlights several aspects worth of discussion. First, although rare, aspergillosis should be considered in the differential diagnosis of dogs with neurological signs and nonspecific changes in CBC or CMP, particularly in German Shepherd dogs. Molecular confirmation is necessary to identify cryptic *Aspergillus* species, particularly when atypical forms are observed, and to plan appropriate treatment.

Finally, our experience confirms that fluconazole should not be the drug of choice in the treatment of systemic aspergillosis and further emphasises the importance of not discontinuing antifungal therapy, even in patients who have been free of clinical signs for a long time.

## Author Contributions

**Talita Bordoni**: conceptualisation, writing – original draft, formal analysis, investigation. **Samuel Okonji**: conceptualisation, formal analysis, writing – original draft, investigation. **Filippo Maria Dini**: formal analysis, writing – review and editing. **Monica Cricca**: formal analysis. **Gualtiero Gandini**: writing – review and editing, supervision. **Monica Caffara**: formal analysis. **Roberta Galuppi**: conceptualisation, writing – review and editing.

## Ethics Statement

The sample was collected during clinical activities performed in accordance with relevant guidelines and regulations, no specific permission was required to perform the sampling.

## Consent

The authors have nothing to report.

## Conflicts of Interest

The authors declare no conflicts of interest.

## Data Availability

All data supporting the present study are showed in the manuscript.
